# The Notch pathway attenuates burn-induced acute lung injury in rats by repressing reactive oxygen species

**DOI:** 10.1093/burnst/tkac008

**Published:** 2022-04-12

**Authors:** Weixia Cai, Kuo Shen, Peng Ji, Yanhui Jia, Shichao Han, Wanfu Zhang, Xiaolong Hu, Xuekang Yang, Juntao Han, Dahai Hu

**Affiliations:** Department of Burns and Cutaneous Surgery, Xijing Hospital, Fourth Military Medical University, Xi’an 710032, China; Department of Burns and Cutaneous Surgery, Xijing Hospital, Fourth Military Medical University, Xi’an 710032, China; Department of Burns and Cutaneous Surgery, Xijing Hospital, Fourth Military Medical University, Xi’an 710032, China; Department of Burns and Cutaneous Surgery, Xijing Hospital, Fourth Military Medical University, Xi’an 710032, China; Department of Burns and Cutaneous Surgery, Xijing Hospital, Fourth Military Medical University, Xi’an 710032, China; Department of Burns and Cutaneous Surgery, Xijing Hospital, Fourth Military Medical University, Xi’an 710032, China; Department of Burns and Cutaneous Surgery, Xijing Hospital, Fourth Military Medical University, Xi’an 710032, China; Department of Burns and Cutaneous Surgery, Xijing Hospital, Fourth Military Medical University, Xi’an 710032, China; Department of Burns and Cutaneous Surgery, Xijing Hospital, Fourth Military Medical University, Xi’an 710032, China; Department of Burns and Cutaneous Surgery, Xijing Hospital, Fourth Military Medical University, Xi’an 710032, China

**Keywords:** Acute lung injury, Notch pathway, Reactive oxygen species, Pulmonary microvascular endothelial cells, Nicotinamide adenine dinucleotide phosphate oxidase 4, Burn

## Abstract

**Background:**

Acute lung injury (ALI) is a common complication following severe burns. The underlying mechanisms of ALI are incompletely understood; thus, available treatments are not sufficient to repair the lung tissue after ALI.

**Methods:**

To investigate the relationship between the Notch pathway and burn-induced lung injury, we established a rat burn injury model by scalding and verified lung injury via lung injury evaluations, including hematoxylin and eosin (H&E) staining, lung injury scoring, bronchoalveolar lavage fluid and wet/dry ratio analyses, myeloperoxidase immunohistochemical staining and reactive oxygen species (ROS) accumulation analysis. To explore whether burn injury affects Notch1 expression, we detected the expression of Notch1 and Hes1 after burn injury. Then, we extracted pulmonary microvascular endothelial cells (PMVECs) and conducted Notch pathway inhibition and activation experiments, via a γ-secretase inhibitor (GSI) and OP9-DLL1 coculture, respectively, to verify the regulatory effect of the Notch pathway on ROS accumulation and apoptosis in burn-serum-stimulated PMVECs. To investigate the regulatory effect of the Notch pathway on ROS accumulation, we detected the expression of oxidative-stress-related molecules such as superoxide dismutase, nicotinamide adenine dinucleotide phosphate (NADPH) oxidase (NOX) 2, NOX4 and cleaved caspase-3. NOX4-specific small interfering RNA (siRNA) and the inhibitor GKT137831 were used to verify the regulatory effect of the Notch pathway on ROS via NOX4.

**Results:**

We successfully established a burn model and revealed that lung injury, excessive ROS accumulation and an inflammatory response occurred. Notch1 detection showed that the expression of Notch1 was significantly increased after burn injury. In PMVECs challenged with burn serum, ROS and cell death were elevated. Moreover, when the Notch pathway was suppressed by GSI, ROS and cell apoptosis levels were significantly increased**.** Conversely, these parameters were reduced when the Notch pathway was activated by OP9-DLL1. Mechanistically, the inhibition of NOX4 by siRNA and GKT137831 showed that the Notch pathway reduced ROS production and cell apoptosis by downregulating the expression of NOX4 in PMVECs.

**Conclusions:**

The Notch pathway reduced ROS production and apoptosis by downregulating the expression of NOX4 in burn-stimulated PMVECs. The Notch–NOX4 pathway may be a novel therapeutic target to treat burn-induced ALI.

Highlights

- This study demonstrated for the first time that the Notch pathway is activated in burn-induced ALI.
- ROS production and cell apoptosis of PMVECs were closely associated with the Notch signaling pathway.
- Activation of the Notch pathway downregulated the expression of ROS and attenuated excessive ROS-induced injury in PMVECs.
- Notch signaling regulated ROS production by modulating the expression of NOX4 rather than NOX2 and SOD1.

## Background

Severe burn injury is a complex pathophysiological process that results in the dysfunction of multiple organs [1–3]. Acute lung injury (ALI) is one of the most critical side effects and is associated with high mortality, especially when the burn area exceeds 30% of the total body surface area (TBSA) [4, 5]. The pathophysiological mechanisms of burn-induced ALI remain incompletely elucidated, although accumulating evidence shows that injury to pulmonary microvascular endothelial cells (PMVECs) plays a key role in the pathogenesis [6, 7]. After a burn injury, pulmonary microvascular lesions, combined with recruited leukocytes, contribute to the production of reactive oxygen species (ROS) [8, 9], which then damage PMVECs and the endothelial barrier [10, 11]. Excessive ROS affect lipids, proteins and DNA and promote irreversible oxidative stress in endothelial cells [12]. Furthermore, oxidative stress in endothelial cells enhances apoptosis and facilitates barrier dysfunction and lung injury [13]. Therefore, ROS production plays a central role in burn-induced ALI [14]. However, the precise molecular mechanisms of ROS production and scavenging in burn-induced injury are not fully understood.

In general, the balance of the production and scavenging of ROS depends on nicotinamide adenine dinucleotide phosphate (NADPH) oxidase (NOX) and superoxide dismutase (SOD)-catalyzed reactions and reductive molecules [15, 16]. The activation of NOX induces EC dysfunction via the production of ROS, while SOD scavenges ROS [17]. The NOX family is a group of important molecules responsible for ROS production [15]. NOX2 and NOX4 are responsible for basal ROS accumulation in endothelial cells (ECs), while the expression of NOX4 is 20 times higher than that of NOX2 [18]. In many diseases, oxidative stress leads to the activation of the NOX family and the accumulation of ROS and myeloperoxidase (MPO), while oxidative stress decreases the expression of antioxidative enzymes, such as SOD and glutathione (GSH), leading to further tissue damage [19–22]. Further studies indicate that NOX4, rather than other NOX isoforms, is responsible for cecal ligation and puncture (CLP)-induced ALI, while NOX4 inhibition alleviates CLP-induced oxidative stress and alleviates ALI [23]. In addition, mounting evidence has revealed that NOX4 plays an important role in many diseases by producing ROS, and NOX4 inhibition exerts protective effects in lung, liver and heart disease as well as diabetes [18, 24–27]. However, the roles and mechanisms of NOX isoforms as well as their modulation in ALI demand further exploration.

Interestingly, the activation of the Notch signaling pathway was shown to alleviate oxidative stress in injured tissues [28–31]. The Notch signaling pathway is evolutionarily conserved and plays a critical role in the regulation of cell differentiation, proliferation and apoptosis [32, 33]. Four transmembrane Notch receptors (Notch1–4) and five ligands (Jagged1, 2 and DLL1, 3, 4) have been identified in mammals [34]. After binding to their ligands, Notch receptors are cleaved by γ-secretase and release their active form, the Notch intracellular domain, into the nucleus. In the nucleus, the Notch intracellular domain interacts with the recombination signal binding protein Jκ (RBP-Jκ), which mediates the transcription of downstream target genes, such as Hes1 and Hey [35]. The Notch pathway plays an important role in endothelial cell function and survival by controlling the production and scavenging of ROS and decreasing ROS-induced cell apoptosis [36–39]. Moreover, an interruption of Notch signaling with a γ-secretase inhibitor (GSI) increases ROS production and aggravates cell injury [29]. In line with a protective effect of the Notch pathway, recent studies have demonstrated that the Notch pathway protects against post-burn myocardial damage [28], ischemia/reperfusion injury [40, 41] and hepatocyte injuries [42] by repressing ROS production. In addition, our previous study indicated that Notch signaling inhibition leads to increased intracellular ROS by upregulating NOX4 expression in primary HUVECs [29].

However, whether Notch1 signaling has a protective effect in burn-induced ALI remains unclear. Here, we investigated the role of Notch signaling in controlling the production of ROS in PMVECs and found that the Notch pathway was activated in early burn-induced ALI. Notch pathway suppression led to the accumulation of ROS and aggravated cell apoptosis. In contrast, Notch pathway activation reduced ROS production by suppressing NOX4 and ameliorated oxidative-stress-induced apoptosis. Thus, the Notch–NOX4 axis is a potential therapeutic target to prevent burn-induced ALI.

**Table 1:** Primer sequences employed for reverse transcription-quantitative polymerase chain reaction

**Gene**	**Forward primer**	**Reverse primer**
TNF-α rat	5′-ATACACTGGCCCGAGGGAAC-3′	5′-CCACATCTCGGATCATGCTTTC-3′
IL-1β rat	5′-CCCTGAACTCAACTGTGAAATAGCA-3′	5′-CCCAAGTCAAGGGCTTGGAA-3’
Notch1 rat	5′-CCCATTACATGCCGCTGTTTC-3′	5′-CATCATGCATTCGGGCATC-3′
Hes1 rat	5′-CAACACGACACCGGACAAAC-3′	5′-GGAATGCCGGGAGCTATCTT-3′
NOX2 rat	5′-GCCCAAAGGTGTCCAAGCT-3′	5’-TCCCCAACGATGCGGATAT-3′
NOX4 rat	5′-GACTTTACAGGTATATCCGGAGCAA-3′	5′-TGCAGATACACTGGGACAATGTAGA-3′
P47 rat	5′-AGCCCTGACTCAAAGGACAAT-3′	5′-TACCCGTGGAGAGAAACCCA-3′
SOD1 rat	5′-AGCATGGGTTCCATGTCCATC-3′	5′-AGCCACATTGCCCAGGTCTC-3′
GAPDH rat	5′-GGCACAGTCAAGGCTGAGAATG-3′	5′-ATGGTGGTGAAGACGCCAGTA-3′
		
NOX4 human	5′-GTTTCAAAGCTGGTCTGCCATTCTA-3′	5′-GATGAAGCCCTGCAGAAGCAA-3′
P47 human	5′-GGGGCGATCAATCCAGAGAAC-3′	5′-GTACTCGGTAAGTGTGCCCTG-3′
Hes1 human	5′-AAAGACGGCCTCTGAGCAC-3′	5′-GGTGCTTCACAGTCATTTCCA-3′
NOX2 human	5′-GCCCAAAGGTGTCCAAGCT-3′	5′-TCCCCAACGATGCGGATAT-3′
NOX4 human	5’-GACTTTACAGGTATATCCGGAGCAA-3’	5′-TGCAGATACACTGGGACAATGTAGA-3′
Notch1 human	5′-TGCCAGGACCGTGACAACTC-3’	5′-CACAGGCACATTCGTAGCCATC-3′
β-Actin h/r/m	5′-GTACGCCAACACAGTGCTG-3′	5′-CGTCATACTCCTGCTTGCTG-3′
		
hs-NOX4-si-1	5′-GGGCUAGGAUUGUGUCUAAdTdT-3′	5′-UUAGACACAAUCCUAGCCCdTdT-3′
hs-NOX4-si-2	5′-CCAGGAGAUUGUUGGAUAAdTdT-3′	5′-UUAUCCAACAAUCUCCUGGdTdT-3′
hs-NOX4-si-3	5′-CGAGAUGAGGAUCCUAGAAdTdT-3′	5′-UUCUAGGAUCCUCAUCUCGdTdT-3′

*TNF-α* tumor necrosis factor α, *IL* interleukin, *NOX*, nicotinamide adenine dinucleotide phosphate oxidase, *SOD* superoxide dismutase, *GAPDH* glyceraldehyde phosphate dehydrogenase

## Methods

### Animals

Adult male Sprague–Dawley (SD) rats with an average weight of 200–250 g were obtained from the Experimental Animal Center of Air Force Medical University (Xi’an, China). Rats were housed in a room with controlled temperature (22 ± 2°C) and light/dark cycles (12 h/12 h). Water and standard chow were freely available to the animals. All animal experiments followed the guidelines of the National Institutes of Health Guide for the Care and Use of Laboratory Animals and were approved by the Animal Experimental Ethics Committee of the Air Force Medical University.

A total of 48 SD rats were randomized into two groups: the sham burn group (sham, *n* = 8) and the burn group (burn, *n* = 40). After being anesthetized (1% pentobarbital sodium 5 mL/kg), rats from the burn group were placed in a 30% TBSA template and immersed in 95°C water for 18 s to establish a full-thickness burn model. The rats immediately received 5 mL of normal saline through intraperitoneal injection for resuscitation. Rats were sacrificed under anesthesia at various endpoints after the burn injury (6, 12, 24, 48, 72 h). Blood samples were taken and centrifuged for 15 min at 1000 × g to obtain serum. Lung tissues were collected and divided into three parts for western blot analysis, qRT-PCR analysis and histopathological evaluation. Samples for biochemical analysis were collected and stored at −80°C until further use.

### Real-time reverse transcription polymerase chain reaction

Total RNA was extracted with TRIzol Reagent (Invitrogen, USA) following the manufacturer’s instructions. cDNA was synthesized from 1 μg of total RNA using the Prime Script RT Reagent kit (Takara, China). Real-time polymerase chain reaction (PCR) was performed using specific primers and SYBR Premix Ex Taq II (Takara, China) in a 20 μL volume and run on a Bio–Rad CFX96 Real-Time PCR system (BioRad, Singapore). Threshold cycle values were determined by CFX96 manager software. mRNA expression was normalized to that of the β-actin gene. The primer sequences used in this study are listed in Table 1.

### Histological and immunohistochemical analyses

For histological analysis, samples obtained from groups 24 h after burn injury were fixed in 10% formalin, dehydrated in a graded alcohol series, embedded in paraffin and cut into 4 μm thick sections. Hematoxylin and eosin (H&E) staining was used for histological observations. MPO and 8-hydroxy-2′-deoxyguanosine (8OHdG) immunohistochemistry staining and immunofluorescence staining for 8OHdG, CD31 and Notch1 (Abclonal, Cat. A11525, USA,) were performed according to the manufacturer’s instructions. Images were collected using an FSX100 microscope (Olympus, Japan).

### Lung injury measurement

The entire lungs were collected for lung wet/dry ratio measurement to assess pulmonary edema after the rats were sacrificed at 6, 12, 24, 48 and 72 h. Bronchoalveolar lavage was performed by injecting the lungs with 0.9% saline (0.5 mL) through the main bronchus, and this procedure was repeated three times. The bronchoalveolar lavage fluid (BALF) was centrifuged for 10 min at 1200 rpm. The supernatants were collected and stored at −80°C. Protein concentrations in BALF were determined by a protein detection kit.

### Enzyme-linked immuno sorbent assay (ELISA) detection

Serum levels of ROS, interleukin-1β (IL-1β) and tumor necrosis factor α (TNF-α) were determined by ELISA using commercially available kits from R&D Systems, Inc., Minneapolis, MN.

### Cell culture and stimulation

As previously described, primary PMVECs were isolated from lung tissues in healthy newborn SD rats [42]. Cobblestone morphology and histochemical staining of factor VIII (Zhongshanxinqiao, China) were used to identify PMVECs. Cells were cultured with endothelial cell medium (ECM) in a humidified incubator at 37°C with a 5% CO_2_ atmosphere. PMVECs were seeded in 6-well plates and treated with GSI, dimethyl sulfoxide (DMSO), burn serum or *N*-acetyl-L-cysteine (NAC, a scavenger of ROS), siNOX4 and GKT137831. Cells from passages 3–5 were studied.

### Coincubation studies

The stromal cell line OP9 was used for coincubation studies. First, DLL1, a ligand of Notch1, and green fluorescent protein (GFP) were overexpressed in OP9 cells [43]. DLL1 is expressed in the cytomembrane of OP9 cells, so coculture with OP9-DLL1 could activate Notch1 expression in PMVECs through the binding of ligands to receptors. Then, PMVECs were cocultured with OP9-DLL1 cells to activate the Notch pathway, while they were cocultured with OP9-GFP cells as a control. OP9, OP9-DLL1 or OP9-GFP cells (1 × 10^5^) were seeded in 6-well plates. After cell adherence, PMVECs (5 × 10^5^) were seeded and cultured for 12 h before being exposed to burn serum.

### Transfection of small interfering RNA

Primary PMVECs were plated into six-well plates (100,000 cells/well) and incubated at 37°C for 18 h. Small interfering (si) RNA-NOX4 (final concentration 20 nM) was added to the cells after treatment with RNAiMAX. Cells were collected 72 h after transfection. Nontargeted siRNA and non-transfected cells were used as controls.

### Flow cytometry

PMVEC apoptosis was measured by flow cytometry using an Annexin V and propidium iodide staining kit (BD Pharmingen™, Cat: 556547, USA) according to the manufacturer’s instructions. The level of intracellular ROS in PMVECs was measured by 2′,7′-dichlorofluorescein (DCFH-DA) (Beyotime, S0033, China) following the recommended protocols. Analyses were performed with flow cytometry (BD FACSAria™ III system, USA). Levels of intracellular ROS were quantified by the mean fluorescence intensity (MFI).

### Western blot

Lung tissue and PMVECs were lysed in radio Immunoprecipitation assay (RIPA) lysis buffer (Beyotime, China) to obtain total proteins. Total extract (30 μg) was subjected to 5–10% sodium dodecyl sulfonate (SDS)-polyacrylamide gel electrophoresis (PAGE) and transferred onto polyvinylidene fluoride (PVDF) membranes. After incubation with 5% nonfat milk, the membranes were probed with the primary antibodies anti-Notch1 (Cell Signaling, #4380, USA), anti-Hes1 (Abcam, ab108937, USA), anti-NOX4 (Epitomics, 3187-1, USA), anti-SOD1 (Epitomics, 2018-1, USA) and anti-cleaved caspase-3 (Cell Signaling, #9664, USA). Glyceraldehyde phosphate dehydrogenase (GAPDH) (Cell Signaling, #5174, USA) was used as the loading control. The membranes were probed with horseradish peroxidase (HRP)-conjugated anti-rabbit-IgG secondary antibodies (Boster Biotechnology, China) and immunoreactive proteins were revealed with enhanced chemiluminescence (ECL, Millipore, USA).

### Statistical analysis

A total of three independent experiments were performed for all analyses. All data are presented as the mean ± SD. Statistical comparisons among groups (≥3) were analyzed using one-way analysis of variance (ANOVA) and the Student–Newman–Keuls *q* test (Graph Pad Prism 8.0), while comparisons among groups (=2) were analyzed by a *t* test (Graph Pad Prism 8.0). Differences with *p* < 0.05 were considered statistically significant.

## Results

### Thirty percent TBSA burn injury triggered acute lung injury in SD rats

**Figure 1.**
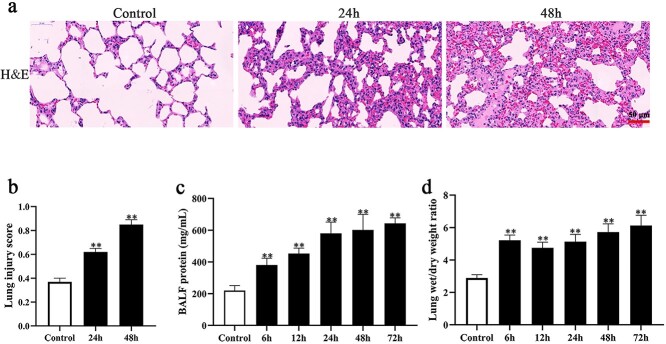
Thirty percent total body surface area (TBSA) burn injury induced acute lung injury. (**a**) Hematoxylin and eosin (H&E) staining of lung tissue in burn injury (24 and 48 h) and control rat, scale bar = 50 μm. (**b**) Lung injury score of burn injury (24 and 48 h) and control rat, ^*^^*^p < 0.01. (**c**) Bronchoalveolar lavage fluid (BALF) protein concentration of burn injury rat (control, 6, 12, 24, 48 and 72 h), ^*^^*^p < 0.01. (**d**) Lung wet/dry weight ratio of burn injury rat (control, 6, 12, 24, 48 and 72 h), ^*^^*^p < 0.01

A total of 40 SD rats underwent 30% TBSA burn injury and were sacrificed under anesthesia at various endpoints after the burn injury (6, 12, 24, 48, 72 h). Lung tissues were collected for H&E staining and injury scoring via lung injury score, BALF and wet/dry ratio analyses. The results indicated that compared with the control group, alveolar capillaries were swollen and congested, and the alveolar cavity was bleeding, accompanied by the infiltration of inflammatory cells in the burn group, particularly at 48 h (Figure 1a). As indicated in Figure 1b, the lung injury score in the burn group was significantly higher than that in the control group. The protein concentration in BALF was detected by a bicinchoninic acid (BCA) kit. The results showed that the protein concentrations in BALF were increased in the burn group compared with the control group (Figure 1c). Figure 1d shows that the wet/dry ratio in the burn group was increased compared with that in the control group at the different endpoints. The above results indicated that 30% TBSA burn injury induced ALI in SD rats.

**Figure 2.**
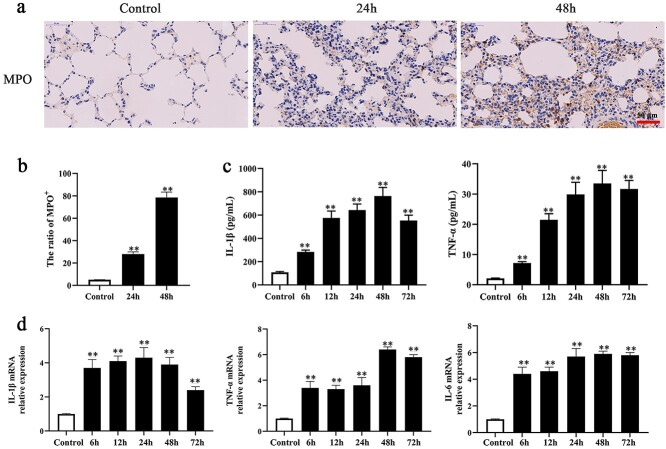
Inflammatory response was involved in burn-induced ALI. (**a**) MPO immunohistochemical staining in burn injury (24 h and 48 h) and control rat, scale bar = 50 μm. (**b**) Quantitative analysis of MPO expression, ratio of (MPO+) = (MPO positive stained cell)/(all cells in each filed of scope) × 100%, ^*^^*^p < 0.01. (**c**) ELISA results of expression of IL-1β and TNF-α in burn injury rat serum (control, 6, 12, 24, 48, and 72 h), ^*^^*^p < 0.01. (**d**) PCR results of mRNA expression of inflammatory factors IL-1β, TNF-α, and IL-6 in burn injury rat lung tissue (control, 6, 12, 24, 48, and 72 h), ^*^^*^p < 0.01. *ALI* acute lung injury, *PCR* polymerase chain reaction, *MPO* Myeloperoxidase, *IL-1β* interleukin-1β, *TNF-α* tumor necrosis factor α

**Figure 3.**
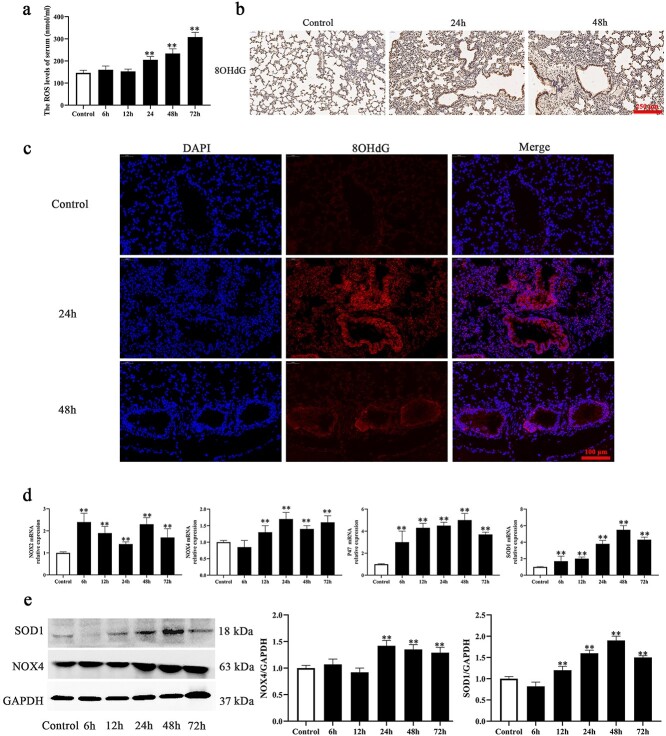
Oxidative stress-related molecules were involved in burn-induced ALI. (**a**) ROS kit results of ROS accumulation in burn injury rat serum (control, 6, 12, 24, 48, and 72 h), ^*^^*^p < 0.01. (**b**) Immunohistochemical staining results of 8OHdG in burn injury (24 h and 48 h) and control rat, scale bar = 250 μm. (**c**) Immunofluorescence staining for 8OHdG in burn injury rat lung tissue (control, 24, and 48 h), blue: DAPI and red: 8OHdG, scale bar = 100 μm. (**d**) PCR results of mRNA expression of oxidative stress-related molecules (NOX2, NOX4, P47, and SOD1) in burn injury rat lung tissue (control, 6, 12, 24, 48, and 72 h), ^*^^*^p < 0.01. (**e**) Expression and analysis of SOD1 and NOX4 in burn injury rat lung tissue (control, 6, 12, 24, 48, and 72 h) detected by western blot, ^*^^*^p < 0.01. *ALI* acute lung injury, *PCR* polymerase chain reaction, *DAPI* 4′, 6-diamidino-2-phenylindole, *GAPDH* glyceraldehyde phosphate dehydrogenase, *8OHdG* 8-hydroxy-2′-deoxyguanosine, *SOD1* superoxide dismutase 1

**Figure 4.**
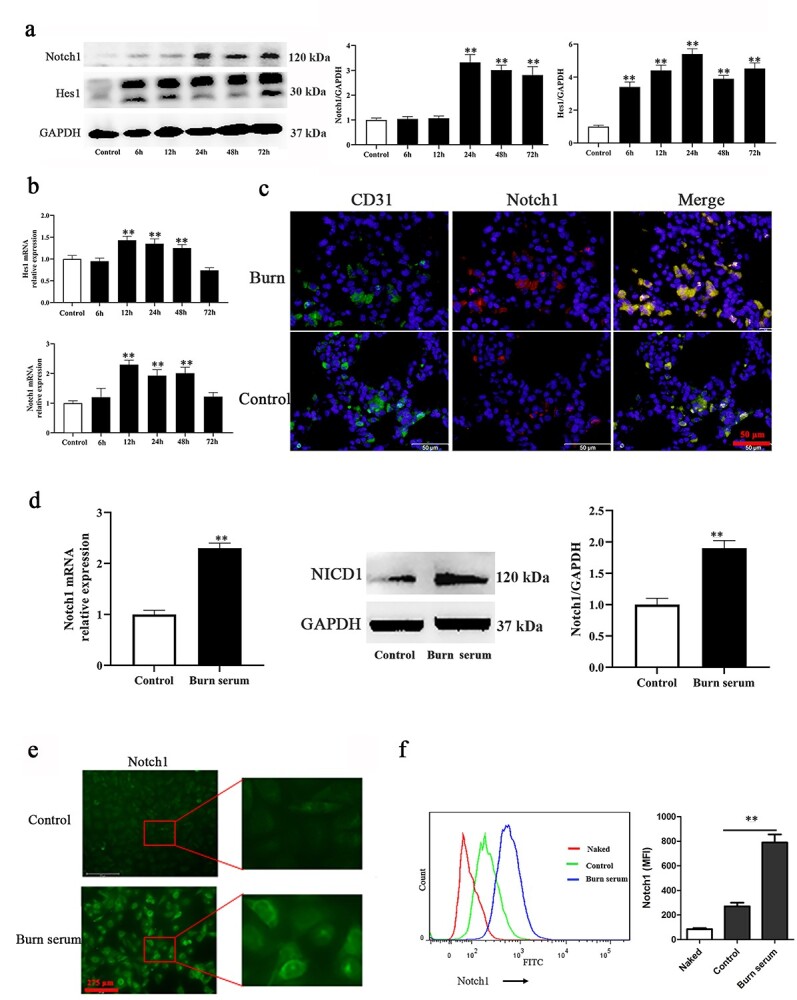
Burn injury activated Notch1 in rat lungs and primary PMVECs. (**a**) Protein expression and analysis of Notch1 and Hes1 in burn injury rat lung tissue (control, 6, 12, 24 and 48 h), ^*^^*^p < 0.01. (**b**) mRNA levels of Notch1 and Hes1 in burn injury rat lung tissue (control, 6, 12, 24 and 48 h), ^*^^*^p < 0.01. (**c**) Immunofluorescence double staining for CD31 and Notch1 in burn injury rat lung tissue (control, 24 and 48 h), green: CD31, red: Notch1 and blue: DAPI; scale bar = 50 μm. (**d**) The mRNA levels, protein expression and analysis of Notch1 in PMVECs challenged with burn serum detected by RT-qPCR and western blot, ^*^^*^p < 0.01. (**e**) Immunofluorescence staining of Notch1 in burn serum stimulated PMVECs and control, scale bar = 175 μm, inset amplification x4. (**f**) Flow cytometry results of Notch1 fluorescence intensity in burn serum stimulated PMVECs and control, ^*^^*^p < 0.01. *PMVECs* pulmonary microvascular endothelial cells, *DAPI* 4′, 6-diamidino-2-phenylindole, *GAPDH* glyceraldehyde phosphate dehydrogenase

### Inflammatory response was involved in burn-induced ALI

The inflammatory response after burn injury was detected by MPO immunohistochemical staining, ELISA and PCR detection of inflammatory factors (IL-1β and TNF-α) in lung tissues and serum. The results showed that MPO expression in neutrophils was significantly promoted 24 and 48 h after burn injury (Figure 2a, b). ELISA results indicated that the expression of the inflammatory cytokines IL-1β and TNF-α in serum increased from 6 to 72 h after burn (Figure 2c). Similarly, as shown in Figure 2d, IL-1β, TNF-α and IL-6 mRNA expression in lung tissue was increased significantly at 6–72 h after burn. These results indicated that the inflammatory response was involved in burn-induced ALI.

**Figure 5.**
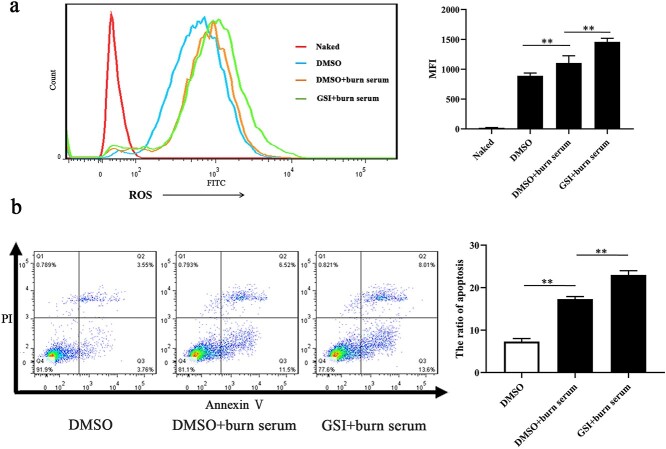
Suppression of Notch signaling led to elevated intracellular ROS and cell apoptosis in primary PMVECs challenged with burn serum. (**a**) Fluorescence intensity and analysis of ROS in primary PMVECs treated with DMSO, DMSO + burn serum, and GSI + burn serum, untreated PMVECs as the control, ^*^^*^p < 0.01. (**b**) Apoptosis and analysis in primary PMVECs treated with DMSO, DMSO + burn serum, and GSI + burn serum, untreated PMVECs as the control. Q2 + Q3 represents apoptosis, ^*^^*^p < 0.01. *PMVECs* pulmonary microvascular endothelial cells, *GSI* γ-secretase inhibitor, *ROS* reactive oxygen species, *DMSO* dimethyl sulfoxide, *MFI* mean fluorescence intensity

### Oxidative-stress-related molecules were involved in burn-induced ALI

Excessive ROS accumulation and impaired antioxidant ability are crucial reasons for lung injury in acute respiratory distress syndrome (ARDS) and other diseases. ROS accumulation was detected by a ROS kit. The results indicated that ROS levels in serum were remarkably elevated from 24 to 72 h post-burn (Figure 3a). 8OHdG is a marker of DNA injury resulting from ROS. We detected 8OHdG expression in burn-induced rat lung tissue by immunohistochemical and immunofluorescence staining. The immunohistochemical staining results indicated that there were many more 8OHdG-positive cells in burn-induced lung tissue (24 and 48 h) than in control lung tissue (Figure 3b, and S1a, see online supplementary material). Immunofluorescence staining also indicated that 8OHdG expression was upregulated in the burn group compared with the control group (Figure 3c, and S1b, see online supplementary material). Furthermore, the expression of the oxidative-stress-related molecules NOX4, P47, NOX2 and SOD1 at the mRNA level in the burn group was increased from 12 h to 72 h post-burn compared with the expression in the control group (Figure 3d). In addition, western blotting showed that the expression of NOX4 and SOD1 was increased at 24 and 48 h post-burn (Figure 3e). In brief, these results indicated that oxidative-stress-related molecules were involved in burn-induced lung injury.

**Figure 6.**
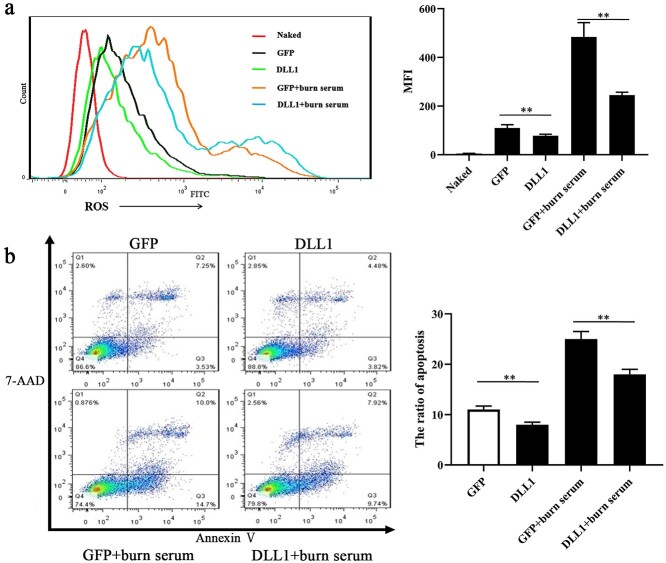
Notch Activation attenuated the elevation of intracellular ROS and cell apoptosis in primary PMVECs challenged by burn serum. (**a**) Fluorescence intensity and analysis of ROS in primary PMVECs co-cultured with DLL1, GFP + burn serum, and DLL1 + burn serum, while PMVECs co-cultured with GFP as the control, ^*^^*^p < 0.01 (**b**) Apoptosis and analysis in primary PMVECs co-cultured with DLL1, GFP + burn serum, and DLL1 + burn serum, while PMVECs co-cultured with GFP as the control, Q2 + Q3 represents apoptosis, ^*^^*^p < 0.01. *PMVECs* pulmonary microvascular endothelial cells, *GFP* OP9 cells over-express GFP, *DLL1* OP9 cells over-express DLL1

### Burn injury activated Notch1 in rat lungs and primary PMVECs

To explore whether burn injury affects Notch1 expression, we first assessed the expression of Notch1 and Hes1 after burn injury. As the western blot results show in Figure 4a, Notch1 and Hes1 expression was remarkably increased from 24 h to 48 h post-burn at the protein level, which corresponded to the PCR results at the mRNA level in Figure 4b. To investigate whether Notch1 was activated in PMVECs after burn injury, lung tissue sections were double stained with anti-CD31 (green) and anti-Notch1 (red) antibodies. Immunofluorescence results showed that the number of cells positively stained with both CD31 and Notch1 at 24 h post-burn was greater than that in the sham group (Figure 4c). Subsequently, we successfully isolated primary PMVECs from healthy newborn SD rat lungs and challenged PMVECs with burn serum. Compared with the control, Notch1 expression in burn serum-stimulated primary PMVECs was significantly upregulated at the mRNA level (Figure 4d). Western blot results also confirmed the elevation of Notch1 expression in primary PMVECs exposed to burn serum (Figure 4d). In addition, Notch1 immunofluorescence staining results showed that the expression and nuclear translocation of Notch1 in primary PMVECs exposed to burn serum were significantly increased compared with those in control serum (Figure 4e). Flow cytometry indicated that burn serum treatment increased Notch1 fluorescence intensity in primary PMVECs (Figure 4f). These findings indicated that burn injury activated Notch1 and Hes1 in rat lungs and primary PMVECs.

**Figure 7.**
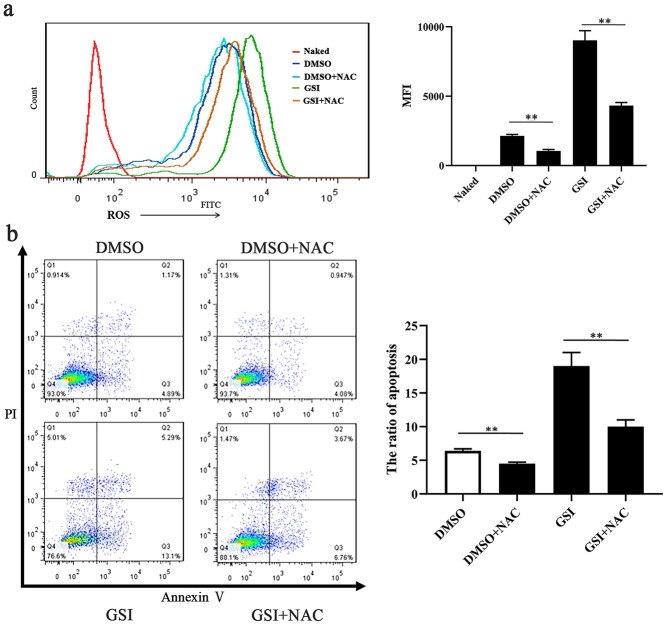
Scavenging ROS by NAC alleviated PMVECs cell apoptosis caused by interruption of Notch signaling. (**a**) Fluorescence intensity and analysis of ROS in primary PMVECs treated with GSI, DMSO + NAC, and GSI + NAC, while DMSO as control, ^*^^*^p < 0.01 (**b**) Apoptosis and analysis in primary PMVECs treated with GSI, DMSO + NAC, and GSI + NAC, while DMSO as control, Q2 + Q3 represents apoptosis, ^*^^*^p < 0.01. *PMVECs* pulmonary microvascular endothelial cells, *GSI* γ-secretase inhibitor, *NAC* N-acetyl-L-cysteine, *PI* propidium Iodide, *GFP* OP9 cells over-express GFP, *DLL1* OP9 cells over-express DLL1

### Suppression of Notch signaling led to elevated intracellular ROS and cell apoptosis in primary PMVECs challenged with burn serum

The above results indicated that the Notch signaling pathway was involved in burn-induced lung injury and primary PMVECs challenged with burn serum, while ROS production was also significantly increased in burn injury. To verify the relationship between the Notch signaling pathway and ROS-induced apoptosis, Notch1 inhibition and activation in primary PMVECs were performed. To inhibit Notch1, PMVECs were exposed to DMSO, DMSO+ 10% Burn serum, and GSI+ Burn serum (an inhibitor of Notch1). At 24 h after treatment, as shown in Figure 5a, the levels of intracellular ROS were significantly increased in the burn serum group. When Notch signaling was interrupted by GSI, ROS levels were further increased (Figure 5a). When challenged with burn serum, the apoptosis rate of PMVECs was upregulated. When PMVECs were pretreated with GSI, cell apoptosis was further aggravated (Figure 5b). Consequently, stimulating primary PMVECs with burn serum led to elevated intracellular ROS and cell apoptosis, while Notch signaling suppression aggravated this effect.

### Notch pathway activation attenuated the elevation of intracellular ROS and cell apoptosis in primary PMVECs challenged with burn serum

To activate Notch signaling, primary PMVECs were cocultured with OP9-Dll1 cells, while PMVECs cocultured with OP9-GFP were considered controls. Compared with control cells (coculture with OP9-GFP), the activation of Notch1 (coculture with OP9-Dll1 cells) reduced intracellular ROS in primary PMVECs (Figure 6a). Additionally, when PMVECs were treated with burn serum, this reduction effect was replicated (Figure 6a). Likewise, the apoptosis rates of primary PMVECs decreased significantly when cocultured with OP9-Dll1 cells, regardless of exposure to burn serum (Figure 6b). That is, when primary PMVECs were cocultured with OP9-Dll1 cells, intracellular ROS and primary PMVEC apoptosis were significantly mitigated. Hence, the activation of the Notch pathway attenuated the burn-serum-induced intracellular ROS accumulation and apoptosis of primary PMVECs. These results suggested that Notch signaling participated in burn-induced ROS accumulation and PMVEC injury.

**Figure 8.**
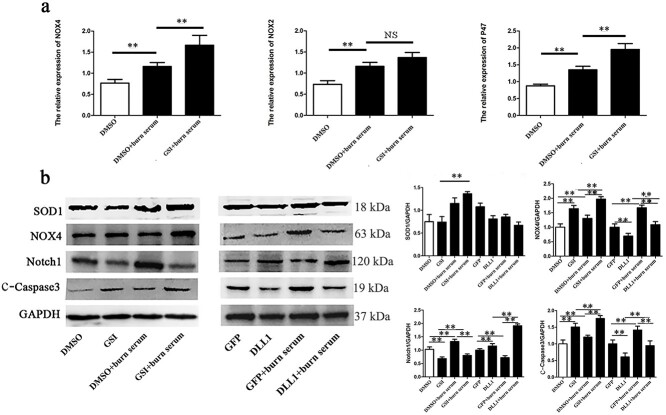
Notch signaling regulated the expressions of NOX4 and c-Caspase-3 rather than NOX2 and SOD. (**a**) mRNA expression of NOX4, NOX2, and P47 in primary PMVECs treated with DMSO + burn serum or GSI + burn serum, while PMVECs treated with DMSO as the control, ^*^^*^  *p* < 0.01, *NS* no significance, *p*>0.05. (**b**) The protein levels and analysis of SOD1, NOX4, Notch1, and c-Caspase3 in primary PMVECs treated with DMSO, GSI, DMSO + burn serum, GSI + burn serum and co-cultured with GFP, DLL1, GFP + burn serum, and DLL1 + burn serum. ^*^^*^p < 0.01; *NS* no significance, *p*>0.05. *PMVECs* pulmonary microvascular endothelial cells, *GSI* γ-secretase inhibitor*, OP9-DLL1* OP9 cells over-expressing DLL1, *OP9-GFP* OP9 cells over-expressing GFP, *c-Caspase3* cleaved caspase-3, *SOD1* superoxide dismutase 1

### Scavenging ROS by NAC alleviated the PMVEC apoptosis caused by the interruption of Notch signaling

To further investigate whether Notch signaling regulated the production of ROS and its protective effect on ROS-induced apoptosis, NAC, a ROS scavenging agent, was applied in subsequent experiments. As shown in Figure 7a, compared with DMSO, the inhibition of Notch signaling (GSI) increased the levels of ROS in PMVECs. To reduce the accumulation of ROS, PMVECs were treated with NAC after exposure to DMSO or GSI. As Figure 7a indicates, treatment with NAC alleviated GSI-induced ROS accumulation. Flow cytometry results showed that scavenging ROS by NAC mitigated the GSI-induced high apoptosis rate in PMVECs (Figure 7b). These results indicated that the interruption of Notch signaling aggravated cell death by enhancing the production of ROS. That is, the activation of Notch signaling exerts a protective effect on PMVECs against ROS-induced injury.

### Notch signaling regulated the expression of NOX4 and cleaved caspase-3 but not NOX2 or SOD1

The above results indicated that Notch signaling alleviated cell death by regulating the production of ROS. We further analyzed oxidative-stress-related molecule expression in PMVECs exposed to burn serum and GSI. PCR results showed that the inhibition of Notch1 by GSI upregulated the expression of NOX4 and P47, while the expression of NOX2 was unchanged in burn-serum-challenged PMVECs (Figure 8a). Then, we detected the protein expression of oxidative-stress-related molecules in PMVECs treated with GSI and DLL1 (Figure 8b). Western blot results showed that SOD1 expression was unaffected by the Notch pathway. Conversely, the expression of NOX4 and cleaved caspase-3 was closely related to the Notch pathway. When Notch signaling was interrupted by GSI, the expression of NOX4 and cleaved caspase-3 was significantly promoted. On the other hand, when Notch signaling was activated by DLL1, the expression levels of NOX4 and cleaved caspase-3 were downregulated. Therefore, Notch signaling may regulate ROS production by modulating the expression of NOX4 rather than the expression of NOX2 and SOD1.

**Figure 9.**
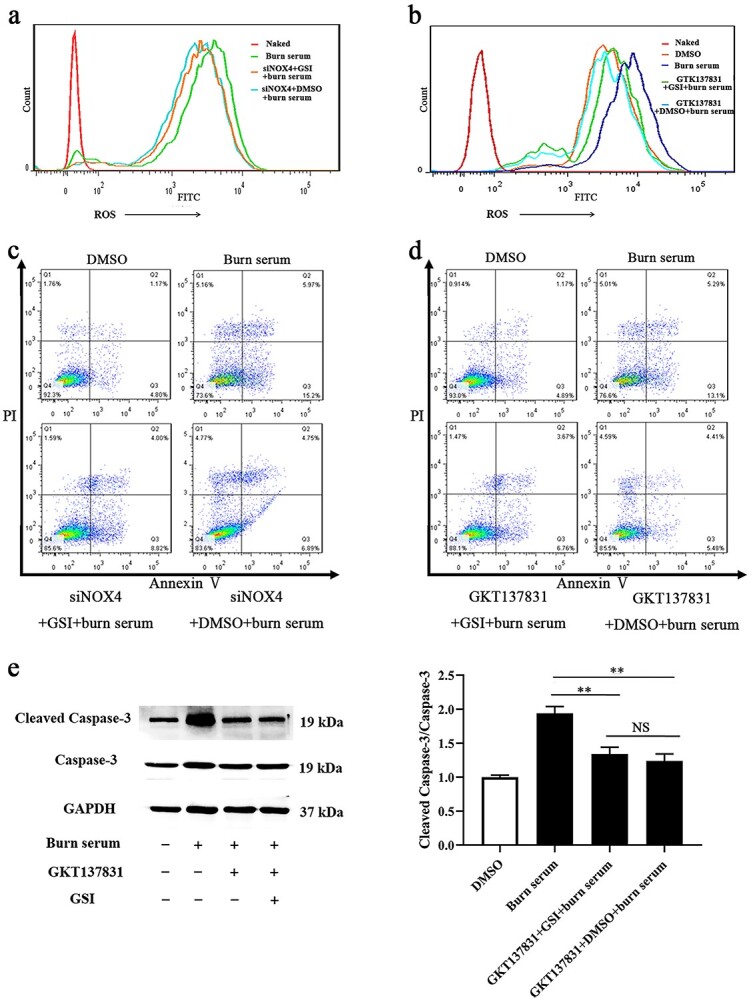
Inhibition of NOX4 eliminated elevated ROS and PMVECs apoptosis caused by Notch suppression. (**a**) Fluorescence intensity of ROS in primary PMVECs treated with Burn serum, siNOX4 + GSI + burn serum, and siNOX4 + DMSO + burn serum. (**b**) Fluorescence intensity of ROS in primary PMVECs treated with Burn serum, GKT137831 + GSI + burn serum, and GKT137831 + DMSO + burn serum, while treated with DMSO as control. (**c**) Apoptosis in primary PMVECs treated with Burn serum, siNOX4 + GSI + burn serum, and siNOX4 + DMSO + burn serum, determined by flow cytometry with PMVECs treated with DMSO as the control. Q2 + Q3 represents apoptosis. (**d**) Apoptosis in primary PMVECs treated with Burn serum, GKT137831 + GSI + burn serum, and GKT137831 + DMSO + burn serum, determined by flow cytometry with PMVECs treated with DMSO as the control. Q2 + Q3 represents apoptosis. (**e**) Expression and analysis of Caspase-3 and Cleaved Caspase-3 in primary PMVECs treated with Burn serum, GKT137831 + GSI + burn serum, and GKT137831 + DMSO + burn serum, determined by western blot with PMVECs treated with DMSO as the control. ^*^^*^p < 0.01. *NS* no significance, GKT137831 inhibitor of NOX4, *PMVECs* pulmonary microvascular endothelial cells, *GSI* γ-secretase inhibitor, *PI* propidium Iodide

### Inhibition of NOX4 eliminated the elevated ROS and PMVEC apoptosis caused by Notch1 suppression

To confirm the modulatory effect of Notch1 on NOX4 in PMVECs, NOX4 was inhibited by siRNA and the inhibitor GKT137831 (Figure S2a, see online supplementary material). Cells were then exposed to burn serum and GSI. As shown in Figure 9a, b, when the expression of NOX4 was inhibited by siNOX4 and GKT137831, the suppression of Notch signaling exerted no effect on intracellular ROS in PMVECs. Statistical analysis revealed that there were no significant differences between the NOX4 inhibition (siNOX4 or GKT137831) + GSI + burn serum group and the NOX4 inhibition (siNOX4 or GKT137831) + burn serum group (Figure S2b, see online supplementary material). In addition, when the expression of NOX4 was inhibited by siNOX4 and GKT137831, apoptosis levels remained invariant when Notch1 was suppressed by GSI (Figure 9c, d, and S2c, see online supplementary material). Moreover, western blot results indicated that when GKT137831 suppressed NOX4, the ratio of cleaved caspase-3/caspase-3 in PMVECs did not change with inhibition of the Notch pathway (Figure 9e). NOX4 inhibition results indicated that when the expression of NOX4 was suppressed, the activation of Notch1 could not exert a protective effect on burn-serum-induced ROS accumulation and apoptosis. Thus, NOX4 may act as the regulatory target of Notch1, which participates in scavenging intracellular ROS and improves cell survival.

## Discussion

Severe burns are often accompanied by fatal complications, of which ALI is one of the earliest and most severe complication [1, 2, 44]. Mounting evidence indicates that PMVEC injury and the accumulation of ROS play an essential role in ALI development [7, 14]. After a burn, the elevation of microvascular permeability results in fluid leakage and abnormalities in microcirculation perfusion. A generalized inflammatory cascade is initiated immediately [13, 45, 46]. Elevated inflammatory mediators trigger ECs to produce excessive ROS [8, 47], which leads to PMVEC injury. Injured PMVECs augment vascular permeability, promoting the infiltration of leukocytes and inflammatory cytokines that worsen and provoke additional lung injury [11, 48]. In this study, we found that the lung tissue was significantly damaged after burn injury, exhibiting pulmonary edema, hemorrhage and inflammatory cell infiltration. Lung injury assessment also indicated that burn injury induced acute lung injury. The detection of the inflammatory response and ROS production indicated that excessive ROS production and the inflammatory response were involved in burn-induced lung injury.

An increasing number of studies have demonstrated that the Notch pathway exerts a protective effect in post-burn myocardial injury or myocardial ischemia reperfusion (MIR) injury [28, 39, 41]. Pei *et al.* indicated that Notch1 played a key role in protection against MI/R injury by reducing oxidative/nitrative stress through the phosphatase and tensin homologue deleted from chromosome 10 gene (PTEN)/Akt pathway [40]. Notch1 plays an essential role in proper EC function and response to injury by specifically regulating downstream molecules [36, 49]. Studies indicate that in primary human umbilical vein endothelial cells, Notch signaling modulates endothelial cell proliferation, migration and adhesion by regulating ROS levels. In our study, we found that Notch1 was activated in the lungs after burn injury. Notch1 expression in PMVECs was also elevated, indicating that Notch signaling might participate in the pathophysiological progress of burn-induced ALI. To explore the mechanisms of Notch signaling in burn-induced ALI, we stimulated primary PMVECs with burn serum to mimic the microenvironment after burn in the subsequent research. Elevated Notch1 expression was also observed in burn-serum-treated PMVECs.

In the pathological process of ALI, excessive ROS has deleterious effects on lipids, proteins and DNA, causing irreversible oxidative stress in cells [50–52]. Furthermore, the accumulation of ROS results in cell apoptosis by inducing the production of inflammatory cytokines [53]. Recent studies have revealed that Notch signaling plays a critical role in the regulation of oxidative stress in cells. The activation of Notch signaling suppresses ROS production, while its inhibition leads to the accumulation of ROS, aggravating cell damage [29]. In intestinal epithelial cells, the activation of the Notch pathway by Jagged-1 significantly reduced ROS generation and apoptosis while increasing cell proliferation [54]. Our previous study indicated that the accumulation of ROS played a crucial role in burn-induced myocardial injury, while the Notch1 pathway exerted a protective effect by repressing ROS through JAK2/STAT3 signaling [28]. In this study, we found that ROS and apoptosis were significantly upregulated when PMVECs were subjected to burn serum. Notch signaling blockade by GSI resulted in remarkably higher levels of ROS and apoptosis. The activation of Notch1 by DLL1 attenuated the apoptosis level of burn-serum-stimulated PMVECs by reducing ROS. Scavenging ROS by NAC alleviated the PMVEC apoptosis caused by the interruption of Notch signaling. These results confirmed that ROS were negatively regulated by Notch signaling and that Notch signaling protected the cell from burn serum challenge by suppressing ROS.

The balance of ROS depends on their production and scavenging. In ECs, the majority of intracellular ROS are produced by the activation of NOX and scavenged through SOD-catalyzed reactions and reductive molecules [55, 56]. The NOX family consists of tissue-specific catalytic subunits NOX1–5 [42]. NOX2 and NOX4 are expressed in ECs, and the expression of NOX4 is 20 times higher than that of NOX2 [57, 58]. Emerging studies indicate that NOX4 plays a crucial role in a wide variety of diseases by regulating the expression of ROS [18, 59]. When exposed to excessive oxidative stress, the inhibition of NOX4 contributes to the protection of lung tissue in lung ischemia and reperfusion injury [60]. Hence, some novel therapeutic approaches to oxidative-stress-induced diseases were developed by inhibiting the expression of NOX4 [61–64]. Moreover, the Notch pathway was reported to confer protection to retinal ECs by regulating NOX4 [30]. However, whether NOX4-mediated ALI affects prognosis in patients and the effects of related therapeutic methods need further verification. In this study, we found that inhibition of the Notch pathway in PMVECs challenged with burn serum led to increased expression of NOX4, while NOX2 was unaffected. SOD1 expression was elevated when exposed to burn injury to counteract oxidative stress, but the inhibition or activation of the Notch pathway exerted no effect on SOD1. However, the expression of NOX4 and cleaved caspase-3 was inversely correlated with Notch signaling. Hence, we assumed that the protective effect of Notch1 from excessive ROS relied on the regulation of NOX4. Further NOX4 inhibition studies revealed that the protective effect of Notch1 was abolished when the expression of NOX4 was inhibited. Therefore, we concluded that Notch signaling could exert a protective effect against oxidative stress by downregulating the expression of NOX4. Bioinformatic analyses revealed that there are two RBP-J binding sites and one Hes1 binding site ~700 bp upstream of the transcription start site of the Nox4 gene. However, the precise mechanism by which the Notch pathway regulates the expression of NOX4 is still unclear.

## Conclusions

This study demonstrated for the first time that the Notch pathway was activated in burn-induced ALI. In addition, ROS production and cell apoptosis were closely associated with the Notch signaling pathway. Furthermore, the activation of Notch1 downregulated the expression of ROS and thus attenuated excessive ROS-induced injury by repressing NOX4. Taken together, our results indicate that the Notch pathway is a novel therapeutic target in strategies to protect against burn-induced ALI.

## Abbreviations

ALI: Acute lung injury; BALF: Bronchoalveolar lavage fluid; GSI: γ-secretase inhibitor; H&E: hematoxylin and eosin; IL-1β: interleukin-1β; MFI: Mean fluorescence intensity; MPO: Myeloperoxidase; NAC: *N*-acetyl-L-cysteine; NOX: NADPH oxidase; 8OHdG: 8-Hydroxy-2′-deoxyguanosine; PMVECs: pulmonary microvascular endothelial cells; RBP-J: Recombination signal binding protein Jκ; ROS: Reactive oxygen species; SD: Sprague–Dawley; siRNA: Small interfering RNA; SOD: Superoxide dismutase; TBSA: Total body surface area; TNF-α: Tumor necrosis factor α.

## Supplementary Material

Supplemental_Materials_tkac008

## Data Availability

The data used to support the findings of this study are available from the corresponding author upon request.
