# Supplementary material for: The Notch pathway attenuates burn-induced acute lung injury in rats by repressing reactive oxygen species
Source: Burns Trauma. 2022 Apr 12;10:tkac008. doi: 10.1093/burnst/tkac008 (PMC9014447; doi:10.1093/burnst/tkac008)
Supplement: Supplemental_Materials_tkac008 [file supplemental_materials_tkac008.pdf]

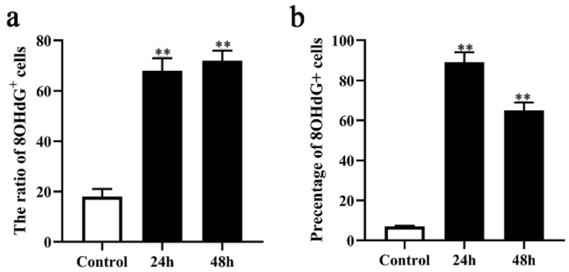

Figure S1. Expression of 8OHdG in burn induced lung injury. (a) Analysis of immunohistochemical staining results of 8OHdG in burn injury (24 h and 48 h) and sham rat, **: P<0.01, compared with control. (b) Analysis of immunofluorescence staining for 8OHdG in burn injury rat lung tissue (0, 24, and 48 h), **: P<0.01, compared with control.

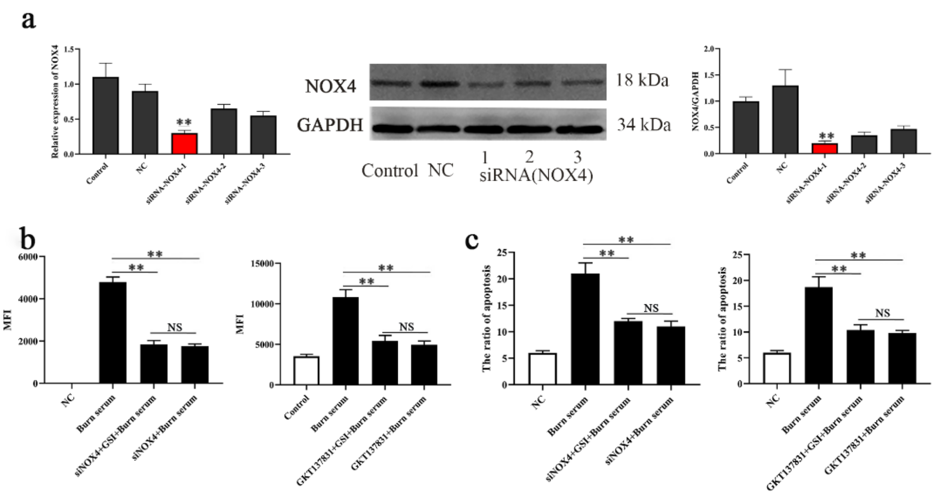

Figure S2. Inhibition of NOX4 eliminated elevated ROS and PMVECs apoptosis caused by Notch suppression. (a) NOX4 mRNA and protein expression and analysis in PMVECs inhibited by different siRNA. (b) Analysis of fluorescence intensity of ROS in primary PMVECs treated with Burn serum, NOX4 inhibition (siNOX4/ GKT137831) + GSI+ Burn serum, and NOX4 inhibition (siNOX4/ GKT137831) + DMSO+ Burn serum, while DMSO as control. **: P<0.01, NS: no significance, P > 0.05. (c) Analysis of apoptosis ratio in primary PMVECs treated with Burn serum, NOX4 inhibition (siNOX4/ GKT137831) + GSI+ Burn serum, and NOX4 inhibition (siNOX4/ GKT137831) + DMSO+ Burn serum, while DMSO as control. **: P<0.01, NS: no significance, P > 0.05.
